# Essential and toxic elements in human milk concentrate with human milk lyophilizate: A preclinical study

**DOI:** 10.1016/j.envres.2020.109733

**Published:** 2020-09

**Authors:** Mariana M. Oliveira, Tânia M.B. Trevilato, Susana I. Segura-Muñoz, Davi C. Aragon, Larissa G. Alves, Martí Nadal, Montse Marquès, José L. Domingo, Jordi Sierra, José Simon Camelo

**Affiliations:** a Department of Pediatrics, Childreńs Hospital, Ribeirão Preto Medical School, University of São Paulo, Ribeirão Preto, São Paulo, Brazil; b Section of Metals and Rare Diseases, Laboratory of Pediatrics, Clinics Hospital of Ribeirão Preto, Ribeirão Preto Medical School, University of São Paulo, Ribeirão Preto, São Paulo, Brazil; c Laboratory of Ecotoxicology and Environmental Parasitology, Ribeirão Preto College of Nursing, University of São Paulo, Ribeirão Preto, São Paulo, Brazil; d Human Milk Bank, Clinics Hospital of Ribeirão Preto, Ribeirão Preto Medical School, University of São Paulo, Ribeirão Preto, São Paulo, Brazil; e Laboratory of Toxicology and Environmental Health, School of Medicine, IISPV, Universitat Rovira I Virgili, Reus, Catalonia, Spain; f Laboratory of Soil Science, Faculty of Pharmacy, Universitat de Barcelona, Catalonia, Spain

**Keywords:** Human milk, Breast milk, Premature infant, Micronutrients, Biomonitoring

## Abstract

Concentrated human milk (HM-concentrate) can be obtained from the simple and inexpensive method of donated breast milk direct lyophilization. A previous study reported that HM-concentrate contains the adequate amount of main macro- and micronutrients for use as a nutritional resource for preterm infants with very low birth weight admitted to neonatal intensive care units. However, further details need to be elucidated about HM-concentrate composition, particularly its content of essential and potentially toxic trace elements. Therefore, this study aimed to determine the concentration of essential and toxic elements in human milk considered baseline (HM-baseline) and HM-concentrate, as well as to quantify changes in concentration of these elements after the HM concentration process. The concentration of Aluminum, Arsenic, Cadmium, Chromium, Iron, Mercury, Manganese, Nickel, Lead, Selenium, Tin, and Thallium was analyzed by inductively coupled plasma-mass spectrometry (ICP-MS). Moreover, Bayesian linear mixed effect models were applied to estimate the mean difference between HM-baseline and HM-concentrate samples. After comparison (HM-concentrate versus HM-baseline), a significant increase in concentration was observed only for Manganese (0.80 μg/L; 95% CrI [0.16; 1.43]) and Selenium (6.74 μg/L; 95% CrI [4.66; 8.86]), while Lead concentration (−6.13 μg/L; 95% CrI [-8.63; −3.61]) decreased. This study provides latest and reliable information about HM composition. After milk concentration by lyophilization, there was a significant increase only in the essential elements Manganese and Selenium. The essential micronutrient content in HM-concentrate was similar or higher than that in preterm mothers' milk, which suggests it is viable for nutritional support of preterm infants. In addition, the low concentrations of potentially toxic elements in HM-concentrate indicates that it is safe for consumption by premature newborns.

## 1 Introduction

Despite rising preterm survival rates, studies show that worldwide, an estimated 9–12% (12.65–16.73 million) of live births are premature (Chawanpaiboon et al., 2019). This significant figure suggests a need for improvement in neonatal nutritional support for preterm infants, especially in low and middle-income countries. Exclusive early enteral nutrition with human milk (HM) is an effective nutritional support strategy for feeding very low birth weight (VLBW) preterm infants. Such practice is associated with lower incidence of necrotizing enterocolitis and neonatal mortality, with reduction in time and cost of hospitalization (Assad et al., 2016; Colaizy et al., 2016; Cortez et al., 2018).

Breast milk provides optimal nutrients and protective factors that enhance immune and gastrointestinal systems, as well as supports long-term neurodevelopment of VLBW preterm infants (Andreas et al., 2015; Brown et al., 2019; Patra et al., 2017; Sammallahti et al., 2017; Walker, 2010). Current recommendations emphasize that preterm newborn infants should receive the mother's milk or HM donated to the Human Milk Bank (HMB), plus commercial fortifiers to improve the milk composition, since milk is the singular nutritional source for infants (Bertino et al., 2013; Colaizy et al., 2012; Dutta et al., 2015; Valentine et al., 2017).

An innovative recent study produced HM-concentrate from a simple and inexpensive method of direct lyophilization of donated breast milk (Oliveira et al., 2019). This concentrated HM demonstrated microbiological safety, acceptable osmolality, and suitable nutritional composition of the main macro- and micronutrients needed by a VLBW preterm infant.

These findings can be translated to clinical trials to test this nutritional resource in preterm infants admitted to neonatal intensive care units. However, there are still not so well-defined aspects of HM-concentrate composition, such as essential and potentially toxic elements content, which could represent a risk for the health of premature infants. Therefore, this study aimed to determine the essential and potentially toxic elements concentration in HM-baseline and HM-concentrate samples, as well as to quantify changes in concentration of these elements after the HM concentration process. The expectation is that our findings confirm and reinforce the viability of HM-concentrate for nutritional support of very low birth weight preterm infants. Furthermore, these findings may have clinical relevance, and update information about mature breast milk composition, which will also be useful for HMB networks worldwide.

## 2 Materials and methods

This cross-sectional study was part of a recently published report approved by the Human Research Ethics Committee of the Clinics Hospital, Ribeirão Preto Medical School - USP (HREC Report No. 738.080), regarding development of a HM-concentrate with HM lyophilizate (Oliveira et al., 2019). Voluntary donors of surplus HM production were informed about the nature of the study, signed a free and informed consent form, and underwent clinical and serological screening. Criteria included being healthy, not smoking more than 10 cigarettes per day, not consuming alcohol or illegal drugs, and providing medical and laboratory exams.

### 2.1 HM sample collection

Donors with a lactation period greater than 15 days were given instructions about massaging and milking their breasts, and about how to withdraw the milk into a sterile, inert glass bottle provided by the HMB. All samples passed through the selection and classification processes recommended by the Brazilian HMB Network (available at: http://www.redeblh.fiocruz.br). A total of 50 samples (≥220 mL) were collected, falling within a standard deviation of 0.36 of the expected average of protein concentration (2.20 g/dL), absolute error value of 0.1, and confidence level of 95%. Surplus mature breast milk with a Dornic acidity value of up to 8°D was included in this study.

### 2.2 HM-baseline and HM-concentrate sample generation

**Fig. 1:**
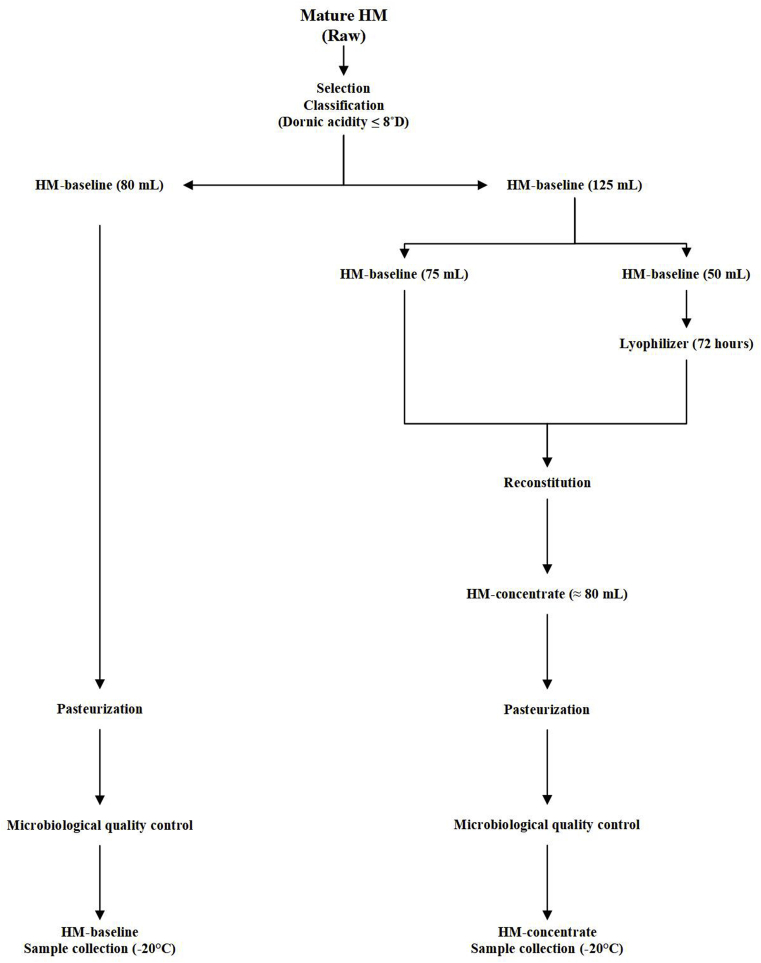
Flowchart of processes applied to HM-baseline and HM-concentrate samples.

Procedures applied to formulate HM-Baseline and HM-Concentrate samples in the HMB at the Clinics Hospital of Ribeirão Preto were recently reported (Oliveira et al., 2019). Briefly, HM-baseline samples were initially separated into two aliquots of 80 and 125 mL each: the 80 mL aliquot was considered the HM-baseline sample. To formulate the HM-concentrate sample, the 125 mL aliquot was further divided into two aliquots of 50 and 75 mL. The 50 mL aliquot was transferred to an inert, sterile glass container and was frozen (−20 °C for 24 h). Subsequently, the frozen sample was lyophilized under vacuum (Lyophilizer L108, LioTop®, São Carlos - SP - Brazil). After 72 h, the lyophilized sample was reconstituted into the other (75 mL) aliquot reserved for the formulation of HM-concentrate. All samples were subjected to pasteurization and microbiological quality control as recommended by the Brazilian HMB Network. In collection tubes, aliquots were separated and immediately frozen (−20 °C) for transport and subsequent analysis. Procedures applied to HM-baseline and HM-concentrate samples are shown in Fig. 1.

### 2.3 Essential and toxic element analysis

Concentrations of essential and potentially toxic elements were analyzed in HM-baseline and HM-concentrate samples at two different timepoints. Preliminary sample preparation was performed in the Laboratory of Pediatrics, Section of Metals and Rare Diseases, Clinics Hospital of Ribeirão Preto, Ribeirão Preto Medical School, University of São Paulo, Ribeirão Preto, São Paulo, Brazil. Samples were thawed at room temperature followed by heating in a water bath (37 °C) and homogenized by sonication for 10 min. Aliquots (1 mL) of the samples were chemically digested with 1 mL of 65% Suprapur nitric acid (Merck KGaA®, Darmstadt, Germany) in hermetic Teflon vessels at room temperature overnight (12 h), followed by heating (80 °C) for 8 h in a hot plate (FANEM®, Model 315 SE, São Paulo, Brazil). Digested samples were diluted by 1:5 using ultrapure water and were sent for analysis by the Laboratory of Toxicology and Environmental Health, School of Medicine, Universitat Rovira i Virgili (Reus, Catalonia, Spain). Following 1:5 dilution with 1% nitric acid (Merck KGaA, Darmstadt, Germany), the concentration of Aluminum (Al), Arsenic (As), Cadmium (Cd), Chromium (Cr), Iron (Fe), Mercury (Hg), Manganese (Mn), Nickel (Ni), Lead (Pb), Selenium (Se), Tin (Sn), and Thallium (Tl) was analyzed by inductively coupled plasma-mass spectrometry (ICP-MS; PerkinElmer® NexION 350D). Reference certificates, duplicates and laboratory blanks were used as quality control to ensure method accuracy and precision. The limit of quantification (LOQ) and limit of detection (LOD) were, respectively: 12.5 and 6.25 μg/L for Al; 0.1 and 0.05 μg/L for As, Cd, and Tl; 2.0 and 1.0 μg/L for Cr; 0.2 and 0.1 μg/L for Hg; 20.0 and 10.0 μg/L for Fe; 1.25 and 0.625 μg/L for Mn, Ni, Pb and Sn; and 2.5 and 1.25 μg/L for Se.

### 2.4 Statistical analysis

Mean values, standard deviation (SD), and minimum and maximum values were used to statistically analyze the essential and toxic element concentrations. Bayesian linear mixed effect models were adjusted using OpenBUGS to estimate mean difference and 95% credible intervals when comparing essential and toxic elements concentrations between HM-baseline and HM-concentrate samples.

## 3 Results and discussion

### 3.1 Characteristics of study population

After selection and classification processes based upon donor inclusion criteria (n = 50 donors), the mean Dornic acid value was 4.34°D and the standard deviation (SD) was 1.59. The mean age, weight, height, and gestational age of the donors was, respectively, 30 years (SD = 6), 67.5 kg (SD = 11.9), 1.63 m (SD = 0.59), and 38.4 weeks (SD = 2.3). All donors reported that they did not smoke or consume alcohol and/or drugs at the time of clinical screening.

### 3.2 Descriptive results

Descriptive statistics (Mean, SD, maximum and minimum values) for essential and potentially toxic elements concentration (μg/L) in HM-baseline and HM-concentrate are summarized in Table 1.

**Table 1:** Descriptive statistics (mean, standard deviation (SD), and maximum and minimum values) of essential and potentially toxic elements concentration (μg/L) in HM-baseline and HM-concentrate (n = 49).

**Element**	**HM-baseline**			**HM-concentrate**		
	**Mean (SD)**	**Min**	**Max**	**Mean (SD)**	**Min**	**Max**
**Al**	211.06 (90.06)	112.30	574.60	202.21 (69.30)	121.00	413.90
**As**	0.29 (0.13)	< LOQ	0.63	0.29 (0.14)	< LOQ	0.80
**Cd**	0.37 (0.19)	< LOQ	0.96	0.35 (0.25)	0.10	1.18
**Cr**	4.39 (1.57)	2.32	8.93	5.65 (5.34)	2.63	29.81
**Fe**	673.55 (264.05)	321.40	1608.90	756.95 (279.39)	313.40	1762.50
**Hg**	0.39 (0.25)	< LOQ	1.68	0.42 (0.20)	< LOQ	1.25
**Mn**	5.07 (1.28)	2.75	9.14	5.88 (1.92)	2.94	15.08
**Ni**	6.34 (3.46)	2.96	25.64	6.06 (3.72)	2.65	20.42
**Pb**	12.79 (8.19)	2.17	41.94	6.69 (3.14)	2.72	19.63
**Se**	7.89 (4.63)	< LOQ	27.60	14.60 (7.16)	< LOQ	27.50
**Sn**	2.78 (1.48)	< LOQ	9.46	3.26 (4.19)	< LOQ	25.01
**Tl**	< LOQ (0.06)	< LOQ	0.30	0.10 (0.12)	< LOQ	0.63

LOQ: Limit of quantification.

One sample was discarded from the statistical analysis due to possible external contamination. Comparative results of essential and toxic elements concentration in HM-baseline and HM-concentrate samples are presented in Table 2.

**Table 2:** Mean difference between essential and potentially toxic elements concentration (μg/L) in HM-baseline (T1) and HM-concentrate (T2) (n = 49).

**Element**	**Comparison**	**Mean difference**	**95% CrI Lower limit**	**95% CrI Upper limit**
**Al**	T2 - T1	−7.99	−39.65	23.67
**As**	T2 - T1	−0.004	−0.05	0.04
**Cd**	T2 - T1	−0.02	−0.11	0.07
**Cr**	T2 - T1	1.25	−0.31	2.70
**Fe**	T2 - T1	72.76	−29.93	171.65
**Hg**	T2 - T1	0.03	−0.05	0.12
**Mn**	T2 - T1	0.80	0.16	1.43
**Ni**	T2 - T1	−0.28	−1.62	1.17
**Pb**	T2 - T1	−6.13	−8.63	−3.61
**Se**	T2 - T1	6.74	4.66	8.86
**Sn**	T2 - T1	0.48	−0.70	1.68
**Tl**	T2 - T1	0.03	−0.01	0.06

T1 – HM-baseline; T2 – HM-concentrate; 95% CrI: Credible Interval 95%.

After comparison between HM-concentrate (T2) and HM-baseline (T1), a significant increase was observed in the concentration of only the essential elements Mn (0.80 μg/L; 95% CrI [0.16; 1.43]) and Se (6.74 μg/L; 95% CrI [4.66; 8.86]), while the concentration of the toxic element Pb (−6.13 μg/L; 95% CrI [-8.63; −3.61]) decreased. There was no significant difference in the concentration of other elements (Al, As, Cd, Cr, Fe, Hg, Ni, Sn, and Tl) between HM-concentrate (T2) and HM-baseline (T1).

### 3.3 General findings

This study evaluated the concentration of essential and potentially toxic trace elements in HM-baseline and HM-concentrate samples and quantified changes in the levels of these elements after the concentration process. A significant increase (HM-concentrate versus HM-baseline) in the concentration of the essential elements Mn and Se was found, while concentration of potentially toxic element Pb decreased. Interestingly, the present study provides updated and reliable information about the composition of mature breast milk (HM-baseline) processed by the Brazilian HMB Network, which should be also useful for HMB networks worldwide.

### 3.4 Essential elements in HM

Premature and low birth weight infants have slightly higher requirements of essential micronutrients such as Cr, Fe, Mn, and Se due to the rapid postnatal growth and development, iatrogenic losses, and limited body storage of these elements. Premature birth interrupts the mother's transfer of these nutrients during the third trimester of gestation. Thus, such infants are at increased risk of developing nutritional deficiencies, such as iron deficiency anemia (Finch, 2015; Harding et al., 2017).

Iron plays a crucial role in the metabolic pathway of energy production, in oxygen transport, and in erythropoiesis. It also aids in the growth and neurodevelopment of preterm infants (Moreno-Fernandez et al., 2019). A recent systematic review indicates that long-term Fe supplementation results in improved Fe levels and a reduction in iron deficiency anemia in VLBW preterm infants (McCarthy et al., 2019). The present study highlights that Fe is a predominant essential micronutrient in HM-baseline and HM-concentrate samples (Table 1). Previous studies conducted in Greece (Leotsinidis et al., 2005), Sweden (Björklund et al., 2012), Chile (Castro et al., 2014), Australia (Mohd-Taufek et al., 2016), Switzerland (Sabatier et al., 2019), and Brazil (Alves Peixoto et al., 2019) detected lower Fe content in breast milk compared to the values found in the present study (Table A1). Furthermore, Fe content in HM samples collected 1–2 months postpartum was higher than in samples collected after 6–7 months and 12 months of lactation (Taravati Javad et al., 2018). Therefore, despite the higher baseline Fe content of the HM-baseline used in the present study, the concentration process maintained the high Fe content (Table 2).

Furthermore, a cross-sectional study demonstrated a positive correlation between Fe and Mn concentration at all stages of lactation (Li et al., 2016). Therefore, like Fe, Mn content also decreases over the lactation stages of colostrum, transitional, and mature milk (Björklund et al., 2012). In addition, Mn concentrations in human milk differs among specific populations (Poland, USA, Argentina, and Namibia; Table A1) (Klein et al., 2017). Interestingly, a study conducted on Brazilian breast milk showed a very low Mn content (Cardoso et al., 2014). However, our study found a higher Mn content in HM-baseline, as well as a significant increase of 0.80 μg/L in HM-concentrate (Table 2). Manganese is an enzymatic cofactor in carbohydrate and lipid metabolism, and also essential micronutrient for growth and bone development (ATSDR, 2012c; Kumar et al., 2017). The higher Mn content in HM-concentrate could positively impact the health of VLBW preterm infants. Likewise, it has been suggested that Fe and Mn content in mothers’ milk does not meet the nutritional requirements of preterm infants (Table A1). Consequently, due to its high Fe and Mn content, we believe that our HM-concentrate may be a viable alternative for the nutritional support of premature infants.

Selenium is another essential micronutrient with a high concentration in HM-baseline and a significant increase in concentration in HM-concentrate (Table 1, Table 2). Studies conducted in Sweden (Björklund et al., 2012), Chile (Castro et al., 2014), Australia (Mohd-Taufek et al., 2016), Switzerland (Sabatier et al., 2019), and Slovenia (Jagodic et al., 2020; Snoj Tratnik et al., 2019) indicate similar Se concentrations to those reported in the present study (Table A1). Significantly, Se has been highlighted as an important element for optimal function of antioxidant defense systems in preterm infants (Tindell and Tipple (2018)). Selenoenzymes, including glutathione peroxidase, protect the body against free radical damage that contributes to risk for prematurity diseases such as bronchopulmonary dysplasia, retinopathy of prematurity, and necrotizing enterocolitis (Finch, 2015). The Se content in HM-concentrate is similar to that in mother's milk of preterm infants. Consequently, it indicates suitability of HM-concentrate for use in premature newborns (Sabatier et al., 2019). This speculation is supported by the fact that the milk from mothers of preterm infants has a mean Se content of 16.1 μg/L, which meets the nutritional requirements of premature newborns (Alves Peixoto et al., 2019). In contrast, micronutrients such as Se and Cr are absent in most commercial human milk fortifiers, suggesting that they do not meet the nutritional needs of premature newborns (Koo and Tice (2018)).

Small doses of the essential nutrient Cr are required for proper energy metabolism (ATSDR, 2012b). The mean Cr level in HM-concentrate was similar to that found in a recent study (Samiee et al., 2019) of Iranian breast milk (Table A1). However, previous surveys conducted in Sweden (Björklund et al., 2012) and Brazil (Cardoso et al., 2014) reported very low Cr content in HM (Table A1). Importantly, Holder pasteurization by thermal treatment applied to HMB, as performed in the present study, does not affect Fe, Mn, and Se content in breast milk (Alves Peixoto et al., 2019; Mohd-Taufek et al., 2016).

### 3.5 Potentially toxic elements in HM

Breast milk is non-invasive biomonitoring matrix of exposures to harmful elements such as Al, As, Cd, Hg and Pb. Toxic organic substances, as well as toxic trace elements, which have been accumulate in the tissues of mothers can cross the mammary glands, exposing newborns to postnatal contamination through HM intake. Potential damage to neonatal growth and development caused by these toxic elements may be severe and permanent (Bansa et al., 2017; Bassil et al., 2018; Bastos et al., 2018; Gil and Hernández (2015); Letinić et al., 2016; Rebelo and Caldas, 2016). Early exposure to excessive amounts of these potentially toxic elements can contribute to long-term adverse health effects, such as neurodevelopmental disorders and damage to immune and respiratory functions (Cao et al., 2016; Heyer and Meredith (2017)). In general, breast milk has a low concentration of potentially toxic elements. Importantly, the concentration of the toxic microelements Al, As, Cd, and Pb decrease rapidly and significantly over the stages of lactation (Chao et al., 2014; Martínez et al., 2019). Thus, it is expected that mature milk used in our HM-concentrate may be safe for the nutritional support of premature newborns.

Aluminum is the most abundant metal in the earth's crust (soil, water, and air), as well as the most frequent and highly concentrated element in breast milk (ATSDR, 2008; Bastos et al., 2018). However, Al concentration in our HM-baseline and HM-concentrate was lower than that reported by studies conducted in Iran (Taravati Javad et al., 2018) and Spain (Martínez et al., 2019) (Table A2). It is important to note that only a small amount of Al enters the infant's body through breastfeeding (ATSDR, 2008). However, the sources of Al exposure may be also contaminated by Pb, As, and Cd (Weidenhamer et al., 2017), where As is the most toxic of these elements.

Exposure of lactating mothers to As may occur through drinking water, as well as via consumption of contaminated rice/cereals, fish/seafood, mushrooms, and poultry (ATSDR, 2007; Bassil et al., 2018). The “Priority List of Substances” released in 2017 by the Agency for Toxic Substance and Disease Registry ranked Arsenic as the potentially most hazardous element to human health. The low As concentration in our HM samples was comparable to other studies performed in Sweden (Björklund et al., 2012), Chile (Castro et al., 2014), USA (Carignan et al., 2015), Turkey (Kılıç Altun et al., 2018), and Slovenia (Jagodic et al., 2020; Snoj Tratnik et al., 2019) (Table A2). However, two studies (Bassil et al., 2018; Klein et al., 2017) showed higher mean As concentrations than reported in the present study (>2 μg/L). Nevertheless, only a negligible amount of As is excreted in breast milk, even in highly exposed mothers (Fängström et al., 2008). Furthermore, it has been emphasized that mixed breastfed babies ingested higher As amounts than exclusive breastfeeding group (Castro et al., 2014). Thus, our innovative proposal based on exclusive breast milk diet could be protective to VLBW against As exposure.

Lead, Hg, and Cd are harmful toxic elements that must be taken into account and analyzed in certain regions, particularly in developing countries, to avoid the risk of exposure to infants (ATSDR, 2019; Klein et al., 2017; Pajewska-Szmyt et al., 2019; Rebelo and Caldas, 2016; Samiee et al., 2019).

Mobilization of Pb stored in mother's bones is the main route of HM contamination, and consequent infant exposure may cause damage to neurological development (Chao et al., 2014). Fortunately, the Pb content in breast milk is low despite high Pb concentration in maternal blood (Baranowska-Bosiacka et al., 2016). The mean Pb concentration in breast milk ranges from 2.0 to 16.8 μg/L, as reported by a major multicenter study conducted by the World Health Organization (WHO) (WHO/IAEA, 1989). Recently, two cross-sectional studies conducted in Iran detected high Pb levels in HM. Gasoline, food, water, dust, and cosmetics (lipstick) were identified as possible contamination sources (Khanjani et al., 2018; Vahidinia et al., 2019). However, the present study found a mean Pb concentration in HM-baseline similar to another Brazilian study (Marques et al., 2013), and the values were within acceptable WHO limits (WHO/IAEA, 1989). Interestingly, the concentration process to produce HM-concentrate resulted in a significant reduction of Pb content compared to HM-baseline (Table 2). A study of lactating Lebanese women found a slightly higher mean Pb concentration compared to the concentration observed in the current survey (Table A2) (Bassil et al., 2018). This study also demonstrated a positive association between pre-pregnancy smoking and Pb and Cd levels in breast milk (Bassil et al., 2018).

Cadmium is found in the earth's crust (air, water, and soil) and is associated with Zn, Pb, and Cu ores (ATSDR, 2012a). Tobacco leaves accumulate high Cd levels, and consequently maternal consumption of tobacco or exposure to tobacco smoke enhanced the risk of infant exposure (ATSDR, 2012a; Bassil et al., 2018). Although smoking during pregnancy increased Cd levels in HM by 37%, there was a non-significant increase associated with the number of smoked cigarettes per day (García-Esquinas et al., 2011). Moreover, an Iranian study detected a higher Cd concentration in breast milk of non-smoking mothers (Khanjani et al., 2018). Cadmium concentrations in breast milk from nursing Brazilian mothers ranged from <0.05 to 7.0 μg/L (Cardoso et al., 2014), while the present study detected even lower Cd concentrations in HM-concentrate samples. In addition, these values do not exceed the WHO reported limit (1 μg/L) (WHO/IAEA, 1989), which is not surprising since Brazil strongly recommends against smoking during prenatal care.

Further, a low level of Ni was found in our HM samples, being the Ni concentration lower than that reported in previous studies conducted in Spain (Martínez et al., 2019) and Iran (Salmani et al., 2016) (Table A2). However, investigations conducted in Sweden (Björklund et al., 2012) and Brazil (Cardoso et al., 2014) found Ni levels in HM even lower than observed in the present study (Table A2). Nickel concentrations in breast milk ranged from 4.9 to 16.1 μg/L according to WHO (WHO/IAEA, 1989). The Agency for Toxic Substance and Disease Registry indicates that the Ni concentration in breast milk is either similar to or less than that in cow's milk-based or soy-based infant formula (ATSDR, 2005). Contaminated water and food, as well as inhalation of tobacco smoke may increase the Ni concentration in breast milk (Salmani et al., 2016). The low Pb, Cd, and Ni concentrations found in this study are possibly due to the non-smoking habits reported by our breast milk donors.

The present study also identified low Hg content in HM samples. Similar to Pb, Hg is excreted in breast milk from exposed mothers, and may affect infant neurodevelopment, causing irreversible damage (ATSDR, 1999; WHO, 2008). A multicenter WHO study identified that the normal range for Hg concentration in HM is 1.4–3.3 μg/L (WHO/IAEA, 1989). Moreover, recent studies have shown that breast milk Hg levels range from 0.5 to 7.0 μg/L worldwide (Cunha et al., 2013; García-Esquinas et al., 2011; Rebelo and Caldas, 2016; Vahidinia et al., 2019). Although some studies have reported a positive correlation between fish consumption and Hg accumulation in breast milk, a recent cross-sectional study showed no association between these factors (Behrooz et al., 2012; Letinić et al., 2016; Vahidinia et al., 2019).

Overall, our study confirms the optimal quality of donated breast milk, since only low concentrations of the potentially toxic elements Al, As, Pb, Cd, and Hg were detected in HM samples. Lastly, studies related to the concentration of Sn and Tl in breast milk remain scarce. A multicenter WHO study reported an Sn concentration ranging from 1.4 to 3.3 μg/L in HM, which is comparable with our findings (WHO/IAEA, 1989). Although prenatal Tl exposure could increase the risk of prematurity and low birth weight (Qi et al., 2019; Wu et al., 2019), Tl content in breast milk has not yet been investigated (WHO/IAEA, 1989).

### 3.6 Study limitations

Limitations of this study include the fact that the dietary patterns of the milk donors were not studied, which could have been integrated with the analysis of breast milk elemental composition. Furthermore, the number of donors was limited number due to the difficulties of collecting samples as human milk donations are scarce. Furthermore, the number of donors was rather limited due to the difficulties of collecting samples, taking into account that human milk donations are scarce. Nevertheless, the results of this investigation provide updated and reliable information about breast milk composition. Furthermore, we demonstrate that the HM lyophilization and concentration processes are safe, since only low concentrations of the potentially toxic elements Al, As, Pb, Cd, and Hg were detected in HM-concentrate samples. However, further studies of HM-concentrate are still necessary in order to assess the safety and tolerability for the effective nutritional support of preterm infants.

## 4 Conclusions

Assessment of essential and toxic elements concentration in HM-concentrate confirms and reinforces the viability of this product specially developed to support the effective nutritional requirements of premature infants. Essential micronutrient content in HM-concentrate was similar to or better than that in preterm mothers' milk, which clearly suggests it can be used for preterm infants. In addition, the low content of toxic elements indicates a low risk for premature newborns. Furthermore, the present study provides updated and reliable information about the composition of mature breast milk (HM-baseline), which should be also useful for HMB networks worldwide.

Phase 1, randomized, controlled, double-blinded clinical trial is underway to assess the safety, tolerability, and initial performance of our HM-concentrate as a nutritional support resource for VLBW infants.

## Author contribution

Mariana M. Oliveira: Conceptualization, Data curation, Formal analysis, Investigation, Methodology, Visualization, Writing - original draft. Tânia M. B. Trevilato: Conceptualization, Data curation, Investigation, Methodology, Resources, ValidationWriting - Writing - review & editing. Susana I. Segura-Muñoz: Data curation, Investigation, Methodology, Resources, Validation and Writing - review & editing. Davi C. Aragon: Formal analysisWriting - Writing - review & editing. Larissa G Alves: InvestigationResources. Martí Nadal: Investigation, Methodology, Resources, ValidationWriting - Writing - review & editing. Montse Marquès: Validation and Writing - review & editing. José L. Domingo: Investigation, Methodology, Resources, Validation and Writing - review & editing. Jordi Sierra: Investigation, Methodology, Resources, Validation and Writing - review & editing. José Simon Camelo Jr: Conceptualization, Funding acquisition, Project administration, Resources and Writing - review & editing.

## Declaration of competing interest

No conflicts of interest.

## Appendix A Supplementary data

Table A.1 Comparison of essential elements concentration (μg/L) in HM-baseline and HM-concentrate with studies carried out in Brazil and worldwide [Mean or *Median* (SD = standard deviation)]. Table A.1

Table A.2 Comparison of potentially toxic elements concentration (μg/L) in HM-baseline and HM-concentrate with studies carried out in Brazil and worldwide [Mean or *Median* (SD = standard deviation)]. Table A.2
