# Supplementary material for: Essential and toxic elements in human milk concentrate with human milk lyophilizate: A preclinical study
Source: Environ Res. 2020 Sep;188:109733. doi: 10.1016/j.envres.2020.109733 (PMC7479503; doi:10.1016/j.envres.2020.109733)
Supplement: Table A.1 — Comparison of essential elements concentration (μg/L) in HM-baseline and HM-concentrate with studies carried out in Brazil and worldwide [Mean or Median (SD = standard deviation)]. [file mmc1.pdf]

Reference	Country	Cr	Fe	Mn	Se
**HM-baseline**	Brazil[A]	4.4 (1.6)	673.5 (264.0)	5.1 (1.3)	7.9 (4.6)
**HM-concentrate**	Brazil[A]	5.6 (5.3)	756.9 (279.4)	5.9 (1.9)	14.6 (7.2)
Leotsinidis et al., 2005	Greece[B]	−	544 (348)	4.8 (3.2)	−
Björklund et al., 2012	Sweden[A]	0.3 (0.3)	339 (134)	3.0 (1.4)	13 (2.6)
Castro et al., 2014	Chile[A]	−	323 (−)	4.9 (−)	15 (−)
		−	341 (−)	4.6 (−)	15 (−)
Mohd-Taufek et al., 2016	Australia[A]	−	230 (93)	1.54 (0.7)	14.4 (3.8)
		−	215 (91)[HP]	1.53 (0.7)[HP]	14.3 (3.6)[HP]
Sabatier et al., 2019	Switzerland[A]	−	440 (260)	−	15.0 (4.2)
		−	360 (230)[PT]	−	14.3 (4.7)[PT]
Alves Peixoto et al., 2019	Brazil[A]	−	*320.2* (−)[HP]	*4.9* (−)[HP]	*7.4* (−)[HP]
		−	*380* (−)[PT]	*4.5* (−)[PT]	*12.6* (−)[PT]
Taravati Javad et al., 2018	Iran[A]	−	1050 (1950)	−	−
		−	530 (420)	−	−
		−	420 (330)	−	−
Klein et al., 2017	USA[A]	−	−	2.7 (1.1)	−
	Namibia[A]	−	−	11.6 (9.8)	−
	Poland[A]	−	−	1.6 (0.9)	−
	Argentina[A]	−	−	7.6 (3.8)	−
Cardoso et al., 2014	Brazil[A]	*2.5* (−)	−	*0.3* (−)	−
Jagodic et al., 2020	Slovenia[A,B]	−	−	−	12.3 (2.6)
		−	−	−	12.4 (4.0)
Snoj Tratnik et al., 2019	Slovenia[A,B,C]	−	−	−	12.6 (−)
Samiee et al., 2019	Iran[A]	5.2 (5.3)	−	−	−

Analysis method: A (Inductively Coupled Plasma – Mass Spectrometry); B (Atomic Absorption Spectrophotometry); C (Atomic Fluorescence Spectrometry). HP: Holder pasteurization of HM; PT: Mother's raw milk of preterm infants.
