# Supplementary material for: Essential and toxic elements in human milk concentrate with human milk lyophilizate: A preclinical study
Source: Environ Res. 2020 Sep;188:109733. doi: 10.1016/j.envres.2020.109733 (PMC7479503; doi:10.1016/j.envres.2020.109733)
Supplement: Table A.2 — Comparison of potentially toxic elements concentration (μg/L) in HM-baseline and HM-concentrate with studies carried out in Brazil and worldwide [Mean or Median (SD = standard deviation)]. [file mmc2.pdf]

Reference	Country	Al	As	Cd	Hg	Ni	Pb	Sn
**HM-baseline**	Brazil [A]	211.1 (90.1)	0.3 (0.1)	0.4 (0.2)	0.4 (0.2)	6.3 (3.5)	12.8 (8.2)	2.8 (1.5)
**HM-concentrate**		202.2 (69.3)	0.3 (0.1)	0.3 (0.2)	0.4 (0.2)	6.0 (3.7)	6.7 (3.1)	3.3 (4.2)
Leotsinidis et al., 2005	Greece[B]	−	−	0.2 (0.1)	−	−	0.5 (0.6)	−
Björklund et., 2012	Sweden[A]	185 (584)	0.5 (0.7)	0.1 (0.1)	–	1.0 (6.5)	1.5 (0.9)	0.4 (0.1)
Castro et al., 2014	Chile[A]	−	0.4 (−)	0.2 (−)	−	−	1.7 (−)	−
		−	0.2 (−)	0.2 (−)	−	−	1.0 (−)	−
Taravati Javad et al., 2018	Iran[A]	180 (180)	−	−	−	−	−	−
		260 (610)	−	−	−	−	−	−
		120 (90)	−	−	−	−	−	−
Klein et al., 2017	USA[A]	−	3.5 (1.1)	−	−	−	0.8 (0.4)	−
	Namibia[A]	−	6.7 (2.5)	−	−	−	2.1 (0.2)	−
	Poland[A]	−	3.9 (1.0)	−	−	−	1.0 (0.3)	−
	Argentina[A]	−	4.5 (1.3)	−	−	−	0.6 (0.4)	−
Cardoso et al., 2014	Brazil[A]	−	−	*0.8* (−)	<0.2 (−)	*1.2* (−)	*0.3* (−)	<*0.1* (−)
Jagodic et al., 2020	Slovenia[A,B]	−	0.6 (0.8)	0.1 (0.0)	0.3 (0.2)	−	0.4 (0.3)	−
		−	0.2 (0.1)	0.1 (0.0)	0.2 (0.2)	−	0.4 (0.5)	−
Snoj Tratnik et al., 2019	Slovenia[A,B,C]	−	0.2 (−)	−	0.1 (−)	−	0.2 (−)	−
Bassil et al., 2018	Lebanon[B]	−	2.4 (1.9)	0.9 (1.2)	−	−	18.2 (13.3)	−
Martínez et al., 2019	Spain[A]	280 (670)	< LOD (−)	20 (30)	30 (10)	40 (40)	30 (10)	10 (0)
Carignan et al., 2015	USA[A]	−	*0.3* (−)	−	−	−	−	−
Kılıç Altun et al., 2018	Turkey[A]	−	<1 (−)	−	−	−	<1 (−)	−
Khanjani et al., 2018	Iran[B]	−	−	8.0 (15.5)	−	−	53.6 (64.9)	−
Vahidinia et al., 2019	Iran[A]	−	−	−	< *LOQ* (−)	−	*38.8* (−)	−
		−	−	−	< *LOQ* (−)	−	*42.3* (−)	−
Marques et al., 2013	Brazil[B]	−	−	−	−	−	12.6 (8.2)	−
		−	−	−	−	−	4.3 (4.0)	−
Salmani et al., 2016	Iran[B]	−	−	−	−	51.0 (7.6)	−	−
Cunha et al., 2013	Brazil[C]	−	−	−	6.5 (6.0)	−	−	−

Analysis method: **A** (Inductively Coupled Plasma – Mass Spectrometry); **B** (Atomic Absorption Spectrophotometry); **C** (Atomic Fluorescence Spectrometry).
